# Genetic evidence for a novel competence inhibitor in the industrially important *Bacillus licheniformis*

**DOI:** 10.1186/s13568-017-0447-5

**Published:** 2017-07-11

**Authors:** Christine Muth, Meike Buchholz, Christina Schmidt, Sonja Volland, Friedhelm Meinhardt

**Affiliations:** 1 Institut für Molekulare Mikrobiologie und Biotechnologie, Westfälische Wilhelms-Universität, Corrensstr. 3, 48149 Münster, Germany; 2 Department of Genomic and Applied Microbiology, Georg-August-University Göttingen, Grisebachstr. 8, 37077 Göttingen, Germany

**Keywords:** *B. licheniformis*, ComI, Competence, Competence inhibitor

## Abstract

Natural genetic competence renders bacteria able to take up and, in case there is sufficient homology to the recipient’s chromosome, integrate exogenously supplied DNA. Well studied in *Bacillus subtilis*, genetic competence is—in several aspects—known to be differently regulated in *Bacillus licheniformis*. We now report on the identification of a novel, chromosomally encoded homolog of a competence inhibitor in *B. licheniformis* (ComI) that has hitherto only been described as a plasmid borne trait in the ancestral *B. subtilis* NCIB3610. Bioinformatical analysis that included 80 *Bacillus* strains covering 20 different species revealed a ComI encoding gene in all of the examined *B. licheniformis* representatives, and was identified in few among the other species investigated. The predicted ComI of *B.* *licheniformis* is a highly conserved peptide consisting of 28 amino acids. Since deletion of *comI* in *B.* *licheniformis* DSM13 resulted in twofold increased transformation efficiency by genetic competence and overexpression resulted in threefold decreased transformability, the function as a competence inhibitor became evident.

**Electronic supplementary material:**

The online version of this article (doi:10.1186/s13568-017-0447-5) contains supplementary material, which is available to authorized users.

## Introduction

Various bacterial species can develop natural genetic competence, a physiological state that enables cells to take up DNA (Dubnau 1999; Johnsborg et al. 2007). The regulatory system governing genetic competence has been studied rather thoroughly in the gram-positive model organism *Bacillus subtilis* (Dubnau 1999; Hamoen et al. 2003; Spizizen 1958). The development of natural genetic competence in *B. subtilis* depends on environmental stimuli such as nutritional limitation and/or cell density (Hamoen et al. 2003). The key transcriptional regulator for developing natural genetic competence in *B. subtilis* is ComK (van Sinderen et al. 1995). Governing cell division, DNA-binding, -uptake, -recombination and -repair, ComK positively controls expression of more than 100 genes; nine genes are negatively affected (Berka et al. 2002; Hamoen 2011).

In contrast to *B. subtilis*, *Bacillus licheniformis* DSM13 carries an insertion element within *comP* rendering ComP, the sensor histidine kinase required for ComX-sensing, inactive (Lapidus et al. 2002). Removing the insertion element (and thereby restoring an active copy of *comP*) resulted in reduced genetic competence (Hoffmann et al. 2010), which clearly differs from *B. subtilis*. Further regulatory differences concern ComS action (Jakobs et al. 2015), as the two ComS homologs identified in *B. licheniformis* did not impact—contrary to *B. subtilis*—the development of genetic competence.

A competence inhibitor (ComI) was identified in the ancestral *B. subtilis* strain NCIB3610 (Konkol et al. 2013). It is encoded on the endogenous 84-kb plasmid pBS32. ComI renders the strain hardly transformable when compared to the frequently used laboratory strain *B. subtilis* 168 (Nijland et al. 2010). Possibly due to curing, pBS32 is absent in the laboratory strains which descend from *B. subtilis* NCIB3610, such as *B. subtilis* 168, *B. subtilis* PY79 or *B. subtilis* JH642 (Konkol et al. 2013; McLoon et al. 2011). When pBS32 was cured from the ancestral *B. subtilis* NCIB3610, transformation efficiencies via genetic competence indeed increased approximately 100-fold; though a similar drastic effect was observed for deletion of *comI*, the knockout of other plasmid-borne genes positively influenced competence as well (Konkol et al. 2013).

In this study, we provide evidence for a ComI homolog within the species *B. licheniformis*. The predicted protein appears to be conserved among *B. licheniformis* species, only rather seldom ComI homologs could be predicted for other *Bacillus* species. Deletion of *comI* has a beneficial effect on the competence mediated transformability of *B. licheniformis* DSM13, whereas overexpression resulted in a decrease of the transformation efficiency.

## Materials and methods

### Bioinformatical and statistical analysis

Analysis of the primary protein structure of ComI was performed with TMBASE (Hofmann and Stoffel 1993). Sequence analysis was performed with BioEdit 7.0.7.0. Evolutionary history was inferred using the Neighbor-Joining algorithm (Saitou and Nei 1987). The evolutionary distances were computed using the Poisson correction method (Zuckerkandl and Pauling 1965) and are in the units of the number of amino acid substitutions per site. The analysis was conducted using MEGA7 (Kumar et al. 2016). Statistical analysis was performed with GraphPad Prism 7.

### Bacterial strains and growth conditions

The strains and plasmids used in this study are listed in Table 1. Bacteria were cultivated at 37 °C in Luria–Bertani (LB) broth unless otherwise stated. Minimal medium contained 6 g Na_2_HPO_4_ l^−1^, 3 g KH_2_PO_4_ l^−1^, 1 g NH_4_Cl l^−1^, 0.5 g NaCl l^−1^, 0.2% (w/v) glucose, 1 mM MgSO_4_, 0.02% (w/v) Casamino Acids, 0.1 mM CaCl_2_, 0.01% (w/v) yeast extract and 0.2 mg MnSO_4_ l^−1^, pH 7.4. Media for uracil auxotrophic strains were supplemented with 10 µg ml^−1^ uracil. Plasmid-carrying *Escherichia coli* strains were grown with ampicillin (100 µg ml^−1^) and *Bacillus* transformants were grown with erythromycin (1 µg ml^−1^), tetracycline (12.5 µg ml^−1^) or kanamycin (2 µg ml^−1^), respectively.

**Table 1:** Bacterial strains and plasmids

Strain or plasmid	Relevant genotype	Source or reference
Strains		
*Escherichia coli* DH5αF‘	*endA1*, *hsdR17* (r_K_^−^, m_K_^+^), *supE44*, *thi*-*1*, *gyrA96*, *relA1*, ∆(*lacIZYA*-*argF*), *U169*, *deoR*, F’Φ(80d*lacZ* M15)	Woodcock et al. (1989)
*Bacillus subtilis* SCK6	BGSC 1A751 derivative, Em^R^, *lacA::*P*xylA*-*comK*	Zhang and Zhang (2011)
*Bacillus licheniformis* DSM13	Wild type	DSMZ, Accession No. AE017333.1
*Bacillus* *subtilis* NCIB3610	Wild type	Branda et al. (2001)
*Bacillus licheniformis* DSM13 ∆*spoIV*	Sporulation-deficient DSM13 derivative	Hoffmann et al. (2010)
*Bacillus licheniformis* MW3.1	DSM13 derivative; Δ*hsdR1,* Δ*hsdR2,* Δ*pyrE*	Hoffmann et al. (2010)
*Bacillus licheniformis* CM1	MW3.1 ∆*comI*::*aphA*	This work
*Bacillus licheniformis* CM2	MW3.1 P_*comI*_::pMUTIN-comI	This work
Plasmids		
pMMcomK	*E. coli*/*Bacillus* shuttle vector, pMM1522 derivative, *B.* *licheniformis* MW3 ComK expression vector, Amp^R^, Tet^R^	Hoffmann et al. (2010)
pUppem	pUCBM20 derivative, P*rpoB*-*upp* fusion, Amp^R^, Em^R^, ori_*E. coli*_	Borgmeier et al. (2012)
pMB03	pUppem derivative, Kan^R^, Em^R^, Amp^R^, ori_*E.coli*_	M. Buchholz, this laboratory
pUE∆comI	pUppem derivative, ∆*comI*::*aphA* substitution cassette	This work
pMUTIN-GFP+	pMUTIN derivative,integrative vector for *Bacillus*, Amp^R^, Em^R^	Kaltwasser et al. (2002)
pMUTIN-comI	pMUTIN-GFP+ derivative, P_*comI*_-GFP fusion, inducible (IPTG) *comI* expression	This work

### Molecular biological techniques

Cloning in *E. coli* was performed essentially as described in Sambrook and Russel (2001). Genomic DNA from *B. licheniformis* was isolated as previously described (Nahrstedt et al. 2004) or by using a commercially available kit (GeneJET Plasmid Miniprep Kit, Thermo Fisher Scientific Inc., Waltham, USA; QuickExtract™ DNA Extraction Solution, Epicentre^®^, Madison, USA). Plasmid DNA was purified with the GeneJET Plasmid Miniprep Kit (Thermo Fisher Scientific Inc., Waltham, USA). For in vitro amplification of DNA, PCR samples (100 µl) contained 200 µM dNTPs, 100 ng template DNA, 1 pmol of each primer and 1 U Taq, Q5 (New England Biolabs GmbH, Frankfurt a.M., Germany) or the Phusion DNA polymerase (Finnzymes Thermo Fisher Scientific Inc., Waltham, USA). Purification of amplified or restriction fragments from gels was performed applying a GeneJET Gel Extraction Kit (Thermo Fisher Scientific Inc., Waltham, USA). Nucleotide sequences were determined by Eurofins Genomics with the didesoxy chain-termination method (Sanger et al. 1977) using the Mix2Seq kit (Eurofins Genomics GmbH, Ebersberg, Germany).

### Vector construction

Primers used in this study were obtained from Eurofins Genomics GmbH (Ebersberg, Germany) and are listed in Additional file 1: Table S1. For disruption of *comI* in *B. licheniformis* MW3.1 the flanking regions of *comI* were amplified; flank A was obtained using the primer pair comI_delA/comI_delA_KpnI and for flank B the primer pair comI_delB/comI_delB_BamHI was applied. For insertion of *aphA*, the gene was amplified from vector pMB03 using the primer pair KanR_A/KanR_B. For the disruption of *comI*, the flanks and *aphA* were fused by SOE-PCR (splicing by overlap extention) (Heckman and Pease 2007), restricted with *Bam*HI and *Kpn*I and cloned into the likewise restricted pUppem vector resulting in plasmid pUE∆comI.

For the P_*comI*_-GFP fusion the promotor region of *comI* was amplified using the primer pair comI13f_KpnI/comI13r_ClaI. The PCR product was subsequently restricted with *Cla*I and *Kpn*I and ligated into the likewise restricted vector pMUTIN-GFP+, resulting in plasmid pMUTIN-comI.

### Transformation

Plasmids were transformed into *E. coli* using the CaCl_2_ mediated method described by Sambrook and Russel (2001) or into *B. subtilis* SCK6 via a transformation protocol developed by Zhang and Zhang (2011). Sequenced vectors were introduced into *B. licheniformis* via induced genetic competence (Hoffmann et al. 2010).

### Natural competence

Transformation efficiencies were investigated by using a 2-step natural competence protocol (Harwood and Cutting 1990; Hoffmann et al. 2010). Cells were grown overnight on LB agar plates and single colonies were inoculated into 3 ml HS medium, which contained 2 g (NH_4_)_2_SO_4_ l^−1^, 14 g K_2_HPO_4_ l^−1^, 6 g KH_2_PO_4_ l^−1^, 1 g Na_3_citrate × 2 H_2_O l^−1^, 0.2 g MgSO_4_ × 7 H_2_O l^−1^, 0.1% (w/v) yeast extract, 0.02% (w/v) Casamino Acids, 0.8% (w/v) l-arginine, 0.04% (w/v) l-histidine, 0.064 g uracil l^−1^ and 0.5% (w/v) glucose. After overnight incubation at 37 °C with vigorous shaking, 1 ml of the starter culture was used to inoculate 20 ml of prewarmed LS medium, containing 2 g (NH_4_)_2_SO_4_ l^−1^, 14 g K_2_HPO_4_ l^−1^, 6 g KH_2_PO_4_ l^−1^, 1 g Na_3_citrate × 2 H_2_O l^−1^, 0.2 g MgSO_4_ × 7 H_2_O l^−1^, 0.1% (w/v) yeast extract, 0.01% (w/v) casamino acids 0.064 g uracil l^−1^, 2.5 mM MgCl_2_ and 0.5% (w/v) glucose. Upon reaching an optical density at 546 nm (OD_546nm_ of 0.9–1), 1 ml of competent cells were transferred to an Eppendorf cup containing 10 µl 0.1 M EGTA and incubated for 5 min at RT. 1 µg chromosomal DNA from *B. licheniformis* DSM13 ∆*spoIV* was added and incubated for 2–3 h in a Thermomixer (Eppendorf AG, Hamburg, Germany) at 37 °C and 600 rpm. The cells were harvested (1 min, max rpm) in a Eppendorf Centrifuge 5424 (Eppendorf AG, Hamburg, Germany) and washed three times with 15 mM NaCl to remove residual uracil. The cells were subsequently plated on M9 minimal medium without uracil. *B. licheniformis* MW3.1 is uracil auxotroph and can therefore not grow on uracil-deficient medium. Therefore, only cells that took up the chromosomal DNA from *B. licheniformis* DSM13 ∆*spoIV* and complemented the ∆*pyrE* locus are able to grow on M9 minimal medium without uracil. CFUs were subsequently determined.

### GeneBank accession numbers

All primary nucleotide sequences used in this work can be found in the GeneBank sequence database of NCBI. The respective accession numbers are listed in Additional file 1: Table S2.

## Results

### Bioinformatical identification of ComI within the genus *Bacillus*

**Fig. 1:**
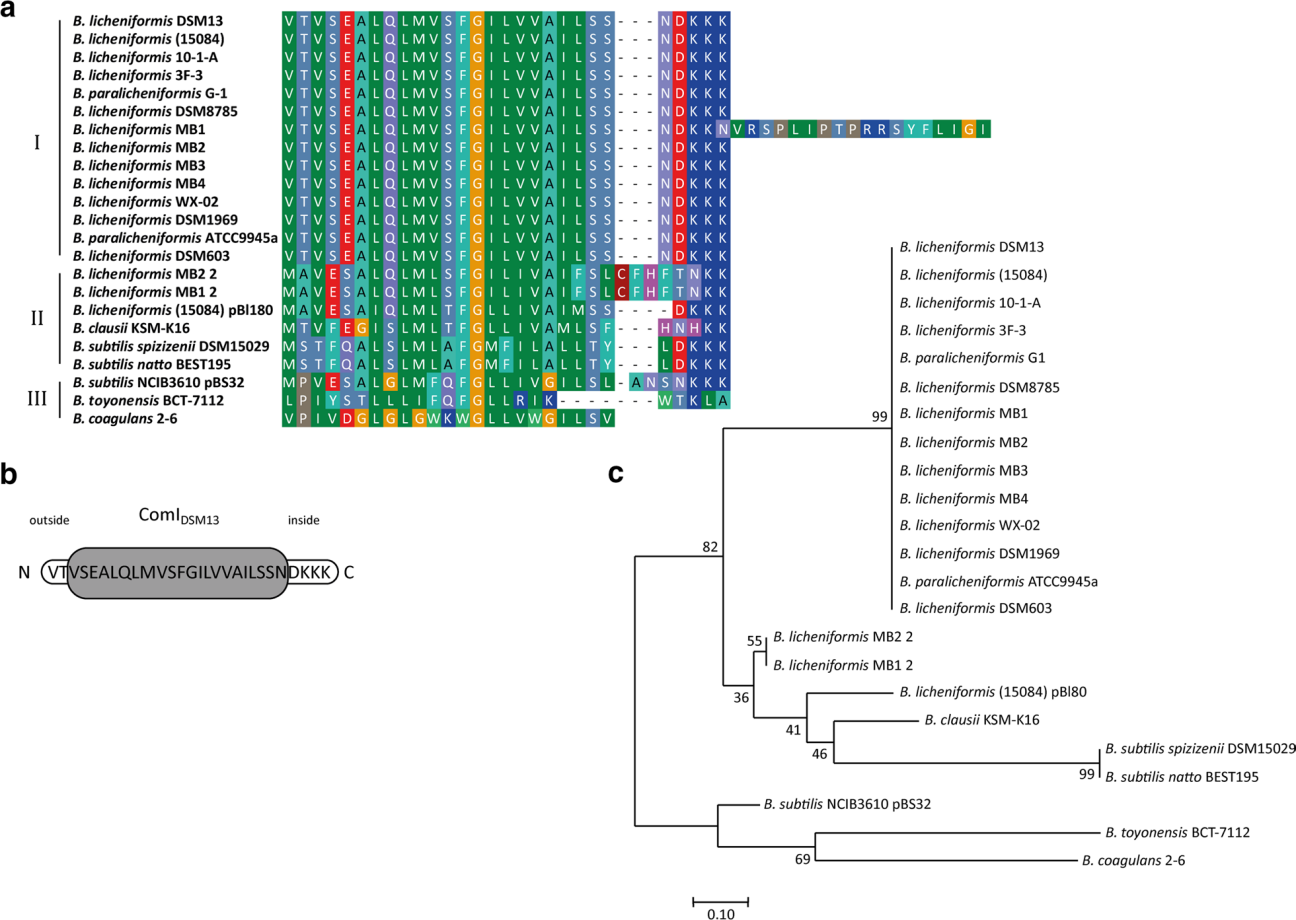
Alignment of ComI homologs predicted for different* Bacillus* species.** a** ComI homologs were identified in 20 strains.* Dark blue boxes* refer to basic aa,* red boxes* to acidic aa. Hydrophobic aa are displayed as* green-shaded boxes* whilst polar, uncharged aa are* bluish violet-shaded*. Glycine is given in* yellow*, cysteine in* maroon* and histidine in* pink*. **b** Primary structure of ComI_DSM13_. Amino acids are given in single-letter code. The N and C termini are indicated as well as the position inside and outside of the cell. The* grey-shaded* protein area indicates the predicted transmembrane helix. **c** Similarity of 23 ComI protein sequences from 20 different *Bacillus* strains. The reliability of the tree was calculated using the bootstrap test (500 replicates) and is shown next to the branches (Felsenstein 1985). The* tree* shows three ComI subgroups

BLAST^®^ Standalone searches disclosed—contrary to the known plasmid-borne ComI of *B.* *subtilis* NCIB3610 (ComI_3610_)—a putative chromosomally encoded homolog in *B. licheniformis* DSM13 (ComI_DSM13_) (Fig. 1a, first line). We were eager to know, whether such chromosomally located gene is present in other *Bacillus* strains and species as well. When bioinformatical analyses were performed, including altogether 80 *Bacillus* strains from 20 different genera (data not shown), a putative *comI* gene was identified for all 14 *B. licheniformis* strains included in the survey, whereas it was rather rarely seen in the other *Bacillus* strains tested (i.e. 4 representatives; see Fig. 1). The predicted ComI of *B. licheniformis* is a highly conserved protein consisting of 28 aa (VTVSEALQLMVSFGILVVAILSSNDKKK). Bootstrap analysis revealed three groups of ComI homologs, with ComI_DSM13_ forming the largest and most conserved group (Fig. 1a, c). Furthermore, a single transmembrane alpha helix could be predicted for ComI_DSM13_ (Fig. 1b). Exemplarily we studied the function of ComI_DSM13_.

### Deletion of *comI* resulted in a twofold increase of transformability

**Fig. 2:**
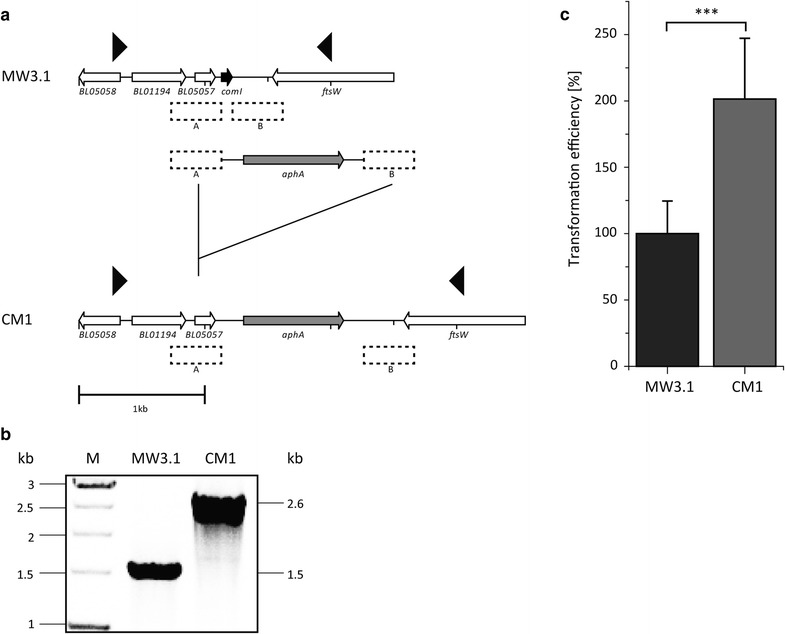
Elimination of* comI* in* B. licheniformis* MW3.1 and its effect on genetic competence.** a** Schematic overview of the genomic *comI* region in *B. licheniformis* MW3.1, the *comI* substitution cassette and the *comI*::*aphA* substitution strain *B. licheniformis* CM1. Open reading frames are shown as* arrows*, primers are depicted as* black triangles* and recombination flanks are given as* rectangles* with* dashed lines*. *comI*, encodes the putative competence inhibitor ComI_DSM13_; *aphA*, kanamycin resistance gene; *ftsW*, cell-division protein; *BL05057*, hypothetical protein of unknown function; *BL05058*, putative DNA-binding protein; *BL01194*, putative DNA-binding protein. **b** Genotypic verification of the *comI*::*aphA* substitution by PCR using the primers comI_seq_1 and comI_seq_4 and subsequent gelelectrophoretic analysis. **c** Transformation frequencies of *B. licheniformis* MW3.1 and *B. licheniformis* CM1 obtained by natural genetic competence with chromosomal DNA of *B. licheniformis* ∆*spoIV* (Hoffmann et al. 2010) applied to restore uracil prototrophy. The transformation frequency for *B. licheniformis* MW3.1 was arbitrarily set as 100%. Data are given as mean ± SD of 3 independent experiments. ***p < 0.001

As ComI_3610_ was already proven to inhibit genetic competence in *B. subtilis* (Konkol et al. 2013), it was tempting to check whether such action is provided by ComI_DSM13_ as well. We therefore used the suicide plasmid pUE∆comI to replace *comI* with the kanamycin resistance cassette *aphA* in the uracil-auxotrophic strain *B. licheniformis* MW3.1, yielding strain *B. licheniformis* CM1 (Fig. 2a). The relevant genetic organization of the strain was examined by PCR analysis (Fig. 2b). The possible effect of the *comI*::*aphA* substitution on natural genetic competence was tested by comparing strain CM1 with its parental strain MW3.1 in transformation experiments. The transformation frequency in *B. licheniformis* MW3.1 was arbitrarily set as 100 (Fig. 2c). The deletion of *comI* had a beneficial effect on the transformability, as CM1 displayed a doubled transformation frequency of 201% ± 4.6, an effect that is nevertheless 50-fold lower than the effect observed in *B. subtilis* NCIB3610, in which the deletion of *comI* resulted in an approximately 100-fold increase of the strain’s transformability (Konkol et al. 2013).

### Recombinant overexpression of *comI* resulted in threefold reduced transformation efficiency

**Fig. 3:**
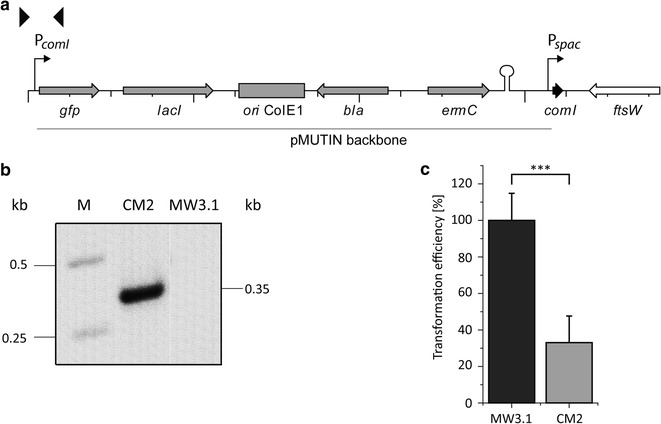
Overexpression of *comI* and its effect on induced genetic competence.** a** Schematic illustration of the genomic region of the pMUTIN-comI integrant *B. licheniformis* CM2. Open reading frames are shown as* arrows*, the direction of which corresponds to the transcriptional orientation. Screening primers are denoted as* black triangles*, promoters are depicted as* angled arrows* and the t1t2t0-terminator is shown as a hairpin-structure. *comI*, encoding the putative competence inhibitor ComI_DSM13_; *ftsW*, encoding a cell-division protein; *gfp*, green fluorescent protein gene; *lacI*, encodes the repressor protein LacI; *ori* ColE1, origin of replication; *bla*, encodes ampicillin resistance; *ermC*, erythromycin resistance gene. **b** Verification of the pMUTIN-comI insertion via PCR with the screening primers comI13f_KpnI and GFPseqr1 and gel electrophoresis. **c** Transformation efficiencies for *B. licheniformis* MW3.1 and *B. licheniformis* CM2 obtained by induced genetic competence using chromosomal DNA of *B. licheniformis* ∆*spoIV* (Hoffmann et al. 2010) to obtain uracil prototrophy (n = 3). Induction of P_*spac*_ was achieved by addition of IPTG to the cultivation medium to a final concentration of 100 µM. Transformation efficiencies for the wild type (MW3.1) were arbitrarily set as 100%. Data are given as mean ± SD of 3 independent experiments. ***p < 0.001

Parallel to the *comI* knockout and the results achieved with strain *B. licheniformis* CM1, we investigated the effect of *comI* overexpression. For such purpose the integrative vector pMUTIN-comI was constructed, in which *comI* is placed under the control of the IPTG-inducible promoter P_*spac*_. Subsequently the construct was established in *B. licheniformis* MW3.1; yielding strain *B. licheniformis* CM2 (Fig. 3a). The correct integration of the expression vector was verified by PCR analysis and gel electrophoresis (Fig. 3b). Possibly due to the fact, that natural genetic competence only renders at maximum 20% of the cells genetically competent (Turgay et al. 1997), experiments with natural genetic competence and *comI* overexpression resulted in transformation frequencies too low for allowing reliable evaluation (data not shown). We therefore performed experiments in which genetic competence was induced by overexpression of *comK* (Hoffmann et al. 2010). Expression of *comI* was achieved by addition of IPTG to the final concentration of 100 µM. Transformation efficiencies for *B. licheniformis* MW3.1 were arbitrarily set as 100%. *B. licheniformis* CM2 yielded only approximately 1/3 (33.06% ± 14.53) of the transformation efficiency compared to *B. licheniformis* MW3.1 (Fig. 3c).

## Discussion

*Bacillus licheniformis* is a close relative to *B. subtilis*. Natural genetic competence, which has been examined thoroughly for *B. subtilis* (Dubnau 1999; Hamoen et al. 2003; Jakobs and Meinhardt 2015; Spizizen 1958), has also been reported for *B. licheniformis* strains (Hoffmann et al. 2010; Jakobs et al. 2014; Leonard et al. 1964; McCuen and Thorne 1971; Thorne and Stull 1966), even though with lower efficiencies than for *B. subtilis* (Jakobs and Meinhardt 2015; Waschkau et al. 2008). Despite the close relationship, major differences in the regulation of genetic competence were seen. While ComP is essential for the development of genetic competence in *B. subtilis* (Weinrauch et al. 1990), *B. licheniformis* DSM13 carries an insertion element in *comP*, which renders ComP inactive (Hoffmann et al. 2010). In contrast to *B.* *subtilis*, the removal of the insertion element led to lower transformation efficiencies (Hoffmann et al. 2010). Furthermore, it became evident that the two *comS* homologs found in *B.* *licheniformis* (ComS1 and ComS2) did not impact genetic competence (Jakobs et al. 2015).

The existence of a functional chromosomal *comI* gene is another remarkable difference between the two species. ComI appears as a highly conserved, 28 aa spanning peptide within *B.* *licheniformis* species, while it is hardly found in *B.* *subtilis*. Indeed, only the plasmid borne, 30 aa peptide-encoding *comI* gene of *B.* *subtilis* NCIB3610 has been reported as a functional competence inhibitor (Konkol et al. 2013). While a *comI* locus has been predicted for *B. subtilis spizizenii* DSM15029 and *B. subtilis natto* BEST195, it remains to be elucidated whether these loci encode for a functional competence inhibitor.

In *B. subtilis* ComI is reported as membrane protein containing a single transmembrane domain, that renders the strain hardly transformable (Konkol et al. 2013). We identified a similar single transmembrane domain in ComI_DSM13_. Interestingly, while the N terminus of ComI_3610_ is predicted to be intracellular (Konkol et al. 2013), an extracellular N terminus is suggested for ComI_DSM13_. Furthermore, glutamine 12 of ComI_3610_ has been described as essential for the protein’s competence inhibiting function, as a G12L substitution rendered the protein inactive for competence inhibition (Konkol et al. 2013). ComI_DSM13_ possesses a serine residue at position 12. Both glutamine and serine are polar, uncharged amino acids. Konkol and colleagues postulated that competence inhibition might be caused by ComI_3610_—directly or indirectly—either separating the energy-providing protein from a transmembrane protein involved in DNA uptake or by preventing the separation of the latter two (Konkol et al. 2013). However, as for ComI_3610_, the mode of competence inhibition needs to be clarified for ComI_DSM13_ as well.

Our results indicate that ComI_DSM13_ has an inhibitory effect on genetic competence in *B. licheniformis*, but does not inhibit competence completely (Hoffmann et al. 2010; Jakobs et al. 2014). The development of genetic competence is a highly sophisticated process, in which the key transcriptional regulator, ComK, controls the expression of competence genes (van Sinderen et al. 1995; Hamoen 2011). The regulation of competence is strictly controlled and, as has been shown before, mainly brought about by deregulation (Hoffmann et al. 2010; Jakobs et al. 2015) and ComI_DSM13_ appears to be a further peptide that controls the development of genetic competence.

As the deletion of *comI* in *B. licheniformis* DSM13 doubled the transformation efficiency rather than increasing the efficiency 100-fold, as for ComI_3610_ (Konkol et al. 2013), the intracellular level of ComI_DSM13_ might be more strictly controlled. However, it must be taken into account that the increased transformation efficiencies described for *B. subtilis* NCIB3610 resulted from curing of the 84 kb endogenous pBS32 plasmid. Even though the deletion of *comI*_3610_ itself increased transformation efficiencies, Konkol and colleagues (2013) demonstrated that the deletion of other genes and gene clusters also had a beneficial effect on the strain’s transformability. pBS32 encodes RapP, a phosphatase that, besides repressing Spo0F activity, also inhibits genetic competence through direct or indirect repression of ComA (Parashar et al. 2013; Omer Bendori et al. 2015). Roughly one-half of the pBS32 located genes encode for phagelike proteins, but the phage-like particles have been shown to be defective and did not kill *B.* *subtilis* (Myagmarjav et al. 2016). ComI_3610_, together with RapP and possibly further, hitherto undetected proteins encoded by pBS32 might promote the intracellular persistence of the plasmid as they, through diminution of genetic competence, prevent the uptake of other, possibly competing plasmids into the cell. The task of plasmid persistence might therefore require a much more drastic way of competence inhibition for ComI_3610_ than is required for the competence-regulating, but not competence-thwarting ComI_DSM13_.

While an interaction with a ComK-induced gene product may prevent ComI_DSM13_ from performing its competence-inhibiting function, regulation of *comI* expression, directly or indirectly through ComK, is conceivable as well. Even though the deletion of *comI* is not crucial for competence development, the deletion greatly improved the transformability and is, thus, a useful tool for enhanced genetic manageability.

## Additional file

**Additional file 1: Table S1.** Oligonucleotides used in this study.** Table S2.** GeneBank accession numbers.

## Abbreviations

~P: phorsphorylated protein
ComI_3610_: competence inhibitor ComI from *Bacillus subtilis* NCIB3610
ComI_DSM13_: competence inhibitor ComI from *Bacillus licheniformis*
*comI*: ComI encoding gene
NCIB: natural collection of industrial bacteria
ComK: key regulator protein for genetic competence
Amp: ampicillin
Kan: kanamycin
Tet: tetracycline
Em: erythromycin
LB: Luria–Bertani (medium)
*aphA*: kanamycin resistance gene
P_*comI*_: promoter of *comI*
P_*spac*_: IPTG inducible promoter
GFP: green fluorescent protein
HS: high salt
LS: low salt
OD: optical density
*spoIV*: gene encoding for the stage IV sporulation protein
CFU: colony forming unit
aa: amino acids
IPTG: isopropyl-β-d-thiogalactopyranoside
G: glutamine
L: leucine
P_*comK*_: promoter of *comK*
SD: standard deviation
BGSC: Bacillus Genetic Stock Center
DSMZ: Deutsche Sammlung von Mikroorganismen und Zellkulturen
