# Supplementary material for: Genetic evidence for a novel competence inhibitor in the industrially important Bacillus licheniformis
Source: AMB Express. 2017 Jul 11;7:149. doi: 10.1186/s13568-017-0447-5 (PMC5503854; doi:10.1186/s13568-017-0447-5)
Supplement: Supplementary file 1 — Additional file 1: Table S1. Oligonucleotides used in this study. Table S2. GeneBank accession numbers. [file 13568_2017_447_MOESM1_ESM.docx]

**Table S1** Oligonucleotides used in this study ^a^

| **Oligonucleotide** | **Sequence (5’ 🡪 3’)** |
| --- | --- |
| comI_delA | AAGATCGCCCTTGATTCCCACAC |
| comI_delA_KpnI | CTCGAT*GGTACC*GCTGACGACAGCCGGGATATTG |
| comI_delB | ATGCTCACCCCCTTTCTCTTCAAGAG |
| comI_delB_BamHI | TGAATCGACT*TGTACA*GTGACAGTATCAGAGGCCTTGCAAC |
| KanR_A | ATCGTCTTGGTGTGGGAATCAAGGGCGATCGATAGTACATAATGGATTTCCTTA |
| KanR_B | CCATCTCTTGAAGAGAAAGGGGGTGAGCATAAATCGGCTCCGTCGATACTATG |
| comI13f_KpnI | TCATGCAGT*GGTACC*AAGGGTTGGCAGCAGTTCGGTTC |
| comI13r_ClaI | AGTCGTACG*ATCGAT*CATGAGTTGCAAGGCCTCTGATAC |
| GFPseqr1 | CAAAGTAGTGACAAGTGTTGG |

^a^: Nonmatching oligonucleotide bases used to create restriction sites are underlined and shown in italics.

**Table S2** GeneBank accession numbers

| **Strain** | **Type of sequence** | **GeneBank accession numbers** |
| --- | --- | --- |
| *B. licheniformis* DSM13 | complete genome | NC_006322 |
| *B. licheniformis* 15084 | *comI* sequence | MF034028 |
| *B. licheniformis* 15084 pBl80 | *comI* sequence | MF034029 |
| *B. licheniformis* 10-1-A | whole genome shotgun sequence | NZ_AJLV00000000 |
| *B. licheniformis* 3F-3 | whole genome shotgun sequence | NZ_JFYM00000000 |
| *B. paralicheniformis* G-1 | whole genome shotgun sequence | AZSK00000000 |
| *B. licheniformis* DSM8785 | *comI* sequence | MF034032 |
| *B. licheniformis* MB1 | *comI* sequence | MF034033 |
| *B. licheniformis* MB1* | *comI* 2 sequence | MF034038 |
| *B. licheniformis* MB2 | *comI* sequence | MF034034 |
| *B. licheniformis* MB2* | *comI 2* sequence | MF034037 |
| *B. licheniformis* MB3 | *comI* sequence | MF034035 |
| *B. licheniformis* MB 4 | *comI* sequence | MF034036 |
| *B. licheniformis* WX-02 | genome | NZ_CP012110 |
| *B. licheniformis* DSM1969 | *comI* sequence | MF034031 |
| *B. licheniformis* ATCC9945a | complete genome | CP005965 |
| *B. licheniformis* DSM603 | *comI* sequence | MF034030 |
| *B. clausii* KSM-K16 | complete genome | NC_006582 |
| *B. subtilis spizizenii* DSM15029 | complete genome | NC_016047 |
| *B. subtilis natto* BEST195 | complete genome | NC_017196 |
| *B. subtilis* NCIB3610 pBS32 | complete plasmid sequence | CP020103 |
| *B. toyonensis* BCT-7112 | complete genome | NC_022781 |
| *B. coagulans* 2-6 | complete genome | NC_015634 |

* second *comI* gene found in the respective strain
